# Domestic and international impacts of rice export restrictions: The recent case of indian non-basmati rice

**DOI:** 10.1016/j.gfs.2024.100754

**Published:** 2024-06

**Authors:** Harold Glenn A. Valera, Ashok K. Mishra, Valerien O. Pede, Takashi Yamano, David Dawe

**Affiliations:** a International Rice Research Institute, Philippines; b Morrison School of Agribusiness, W. P. Carey, School of Business, Arizona State University, Mesa, AZ, 85212, USA; c Asian Development Bank, Philippines; d Bangko Sentral ng Pilipinas Research Academy, Philippines

**Keywords:** Export ban, Domestic rice prices, GARCH, Panel data

## Abstract

This study examines the impact of India's export restrictions on domestic retail rice prices using a dynamic panel GARCH model. The findings suggest that export restrictions are not a sufficient condition to lower domestic prices. Export restrictions are associated with lower retail price volatility in the East Zone. Moreover, the international price transmission to a sample of Asian and African economies shows that all countries are vulnerable, but the degree and kinds of vulnerability differ. Rice exporters appear to be the most susceptible as domestic prices increase in these countries. Rice importers are also vulnerable because of price increases, but the increases are less than in countries where the private sector decides on import quantities.

## Highlights

• We analyze the impact of India's export restrictions on domestic retail rice price.
• Panel GARCH model was employed to daily retail price observations for four zones in India over the period January 1, 2017 to December 18, 2023.
• We find that export restrictions are not a sufficient condition to lower domestic prices.
• Export restrictions are associated with lower rice price volatility in the East Zone.
• Asian exporters and importers and African importers are vulnerable to global rice price fluctuations, but the degree and kinds of vulnerability differ.

## 1 Introduction

India has a history of restricting non-basmati (non-parboiled) rice exports, first in 2007–2011 and again in July 2023. The recent event was triggered by a substantial increase in domestic rice prices in June 2023, when heavy monsoon rains caused significant damage to kharif rice crops. World rice prices rose substantially within the next four weeks after July 20, 2023, when India restricted non-basmati (non-parboiled) rice exports. This rice export restriction coincided with Russia's recent withdrawal from the Black Sea Grain Initiative. Both actions triggered further fears that India's rice export restrictions could threaten global food security, especially because India is the world's largest rice exporter, with a 40% share of the international rice market. Thus, there was a significant shortage in rice inventories and export quantities. There is considerable value in understanding the impacts of India's recent rice export restrictions on developing countries. The sharp increase in world rice prices in August 2023 following such an export policy is an important source of risk in many developing countries whose food security is highly reliant on the world's rice market. This is particularly true for Asian and African importing countries, which have been forced to pay substantially higher prices due to the turmoil in the world's rice market created by India's export restrictions. Both importing and exporting countries face significant increases in retail rice prices and food insecurity.

The theory provides clear and intuitive predictions about the probable effects of export restrictions on staple grains, including rice. For example, studies by Mitra and Josling (2009) and Liefert et al. (2011) warn that export restrictions introduce welfare-reducing price distortions for which domestic farmers lose more than domestic consumers benefit from lower local prices. Mitra and Josling (2009) point out that world prices would remain unaltered and domestic prices would decline if a small open economy implemented export restrictions. However, if the export ban is enforced by a country that is large enough to affect the price of its trading partner, then domestic prices decline while the trading partner's price rises (Abbott, 2011). The expected increase in the prices of staples, such as rice, by trading partners would be even more pronounced, thus increasing global food insecurity. Hence, this directly makes staples less affordable and decreases the available budget for other food groups. Other studies, such as Headey (2011), Martin and Anderson (2011), and Anderson and Nelgen (2012), take a similar route and claim that export restrictions magnify international price fluctuations because of reduced supply to the world market during times of high prices, and thus negatively affect the welfare of people and economies in other countries. However, in the debate surrounding India's rice export ban, a neutral belief is that the export ban would not affect India's reputation as a reliable supplier (Mohanty, 2023).

The literature has so far provided a valuable understanding of the economic impacts of export restrictions on grain markets (see, for example, Fellmann et al. (2014), Pham Van Ha et al. (2015), Diao and Kennedy (2016), Porteous (2017), Aragie et al. (2018), and Sankaranarayanan et al. (2020)). A common feature of those studies is that their empirical work is mostly confined to model-based analysis that offers ex ante insights into the national-level impacts of rice export restrictions on individual countries. However, empirical work that employs this technique fails to assess the immediate impact of export restrictions on the domestic prices in different locations within a country owing to the lack of adequate price data.

With this background, the objective of our study is to conduct an empirical investigation of the recent export restrictions imposed by India on non-basmati (non-parboiled) rice on the level and volatility of domestic rice prices for four zones in India: East Zone, North Zone, South Zone, and West Zone. A shortage of prior investigation of rice price dynamics, either in times of rising prices or following the implementation of a rice trade policy, is because rice price information is typically available at low frequency, such as monthly data. We overcome this critical limitation by using price observations from the database managed by India's Department of Consumer Affairs, whose mandates include, among others, monitoring of prices and availability of essential commodities. The salient feature of the database is that it collects daily retail prices of rice for different states and zones.

This rich data set enables us to contribute to the literature by answering two key research questions. First, is there evidence supporting the reduction in domestic rice prices across different zones since India placed export restrictions on July 20, 2023? Second, to what extent do export restrictions help lower the volatility of domestic prices across different zones? To this end, we apply a dynamic panel GARCH model proposed by Cermeño and Grier (2006). According to Lee (2010), panel GARCH models can provide potential efficiency gains in estimating conditional variance and covariance processes by capturing relevant information on heterogeneity across locations and their interdependence. The current study also provides complementary information on the impact of India's rice export restrictions on world rice prices and the transmission of international prices to domestic rice markets in Asia and Africa by analyzing the cumulative percentage increase in domestic rice prices based on available monthly data. This allows us to examine the degree and kinds of vulnerability of exporting and importing countries to global rice price fluctuations. By shedding additional light on the results obtained from the cumulative percentage increase in the domestic prices of importing and exporting countries, we offer complementary information to the daily rice price analysis mentioned earlier.

The rest of the study is organized as follows. Section 2 discusses the importance of India's rice exports in the international market and previous studies on export restrictions on cereals. Section 3 presents the methodology and data. Section 4 discusses the empirical results. Section 5 presents conclusions and policy implications.

## 2 Background and recent literature

### 2.1 Background on the Indian non-basmati (non-parboiled) rice market

There are two broad categories of Indian rice exports: basmati and non-basmati rice. The non-basmati rice category has six sub-categories: (i) non-basmati white^1^ rice, (ii) broken rice, (iii) parboiled rice,^2^ (iv) rice in the husk of seed quality, (v) other rice in the husk, and (vi) husked (brown) rice. The export restrictions announced by India on July 20, 2023, covered only the export sub-categories broken rice and non-basmati white rice. The latter export sub-category comprises semi-milled or wholly milled, polished or unpolished, and glazed non-basmati white rice. It is important to point out that, for the Indian export restrictions imposed after July 2023, non-basmati white rice export is allowed based on permission granted by the Government of India to other countries to meet their food security needs and based on the request of their government (Sharma, 2023).

**Fig. 1:**
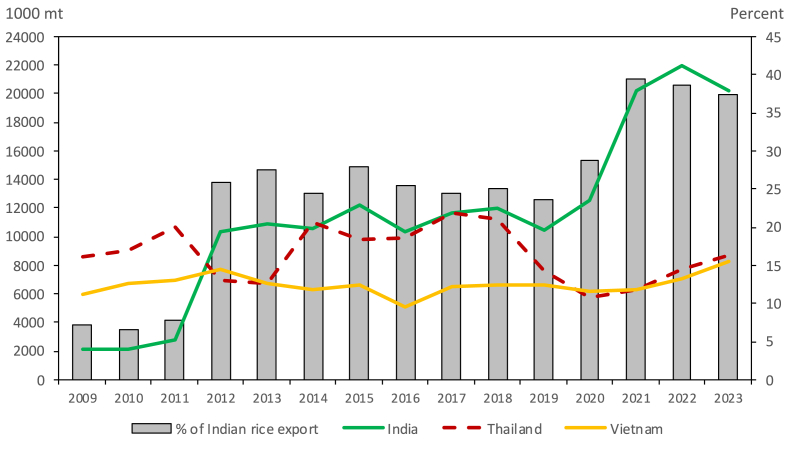
Rice exports of India, Thailand, and Vietnam. *Source of data*: USDA Production, Supply and Distribution (PSD) database.

**Fig. 2:**
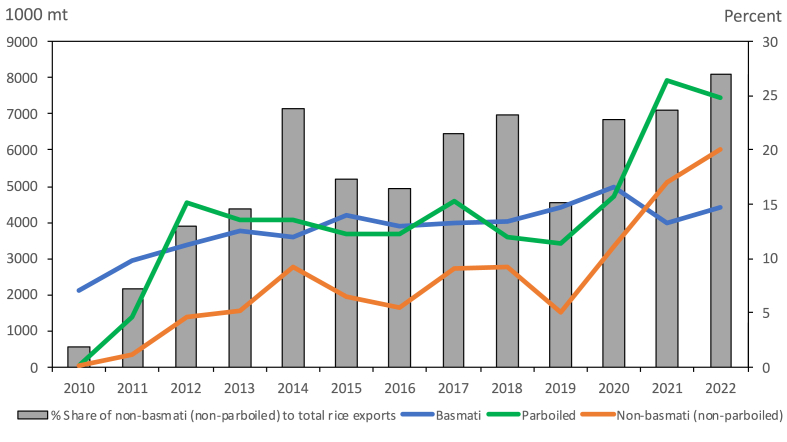
Indian exports of basmati and non-basmati (non-parboiled) white rice. *Source of data:* Trade Data Monitor.

Fig. 1 shows India's performance relative to that of other large rice-exporting countries such as Thailand and Vietnam. India started surpassing Thailand as the largest exporter in the global rice market in 2011 and had a substantial lead in 2019. The status of India as the world's largest rice exporter can be attributed to its capability in capturing the market share of Thailand, whose export volume has decreased since 2017. Looking at the specific types of rice exports in India, Fig. 2 summarizes the export trends of basmati, non-basmati (non-parboiled), and parboiled rice. Non-basmati (non-parboiled) rice has the lowest contribution to total exports among the three categories from 2010 to 2020. However, due to higher production, non-basmati (non-parboiled) rice exports continuously increased from 2020 to 2022. Indeed, the volume of non-basmati (non-parboiled) rice exports during 2021–2022 was higher than that of basmati rice. Non-basmati (non-parboiled) rice exports accounted for more than 20% of India's total exports from 2020 to 2022. It is also worth noting that non-basmati (non-parboiled) rice exports still increased even after the imposition of a 20% export tariff on white non-basmati rice in 2022.

**Fig. 3:**
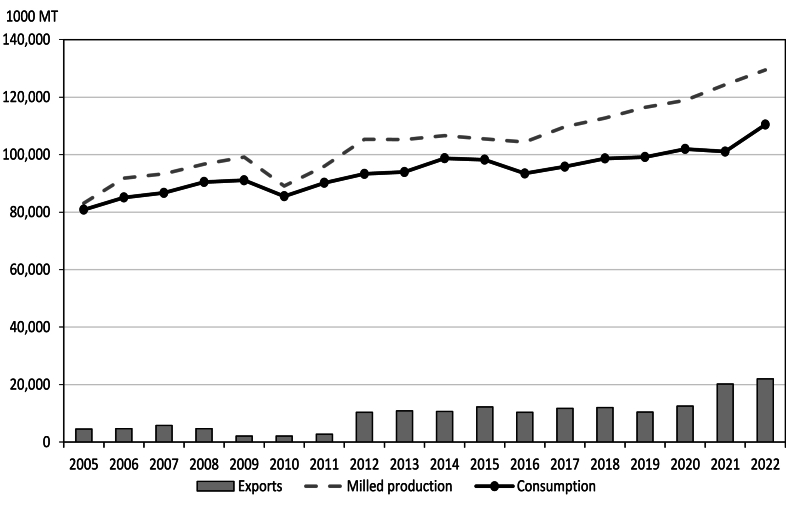
Milled rice production, consumption, and exports of India, 2000–2018. *Source:* Milled rice production and consumption are compiled from USDA Production, Supply and Distribution (PSD) database.

Non-basmati (non-parboiled) rice has not been India's top export, but it is still imported by more than 150 countries worldwide. Table 1 summarizes the major export destinations of Indian non-basmati (non-parboiled) rice in Africa and Asia during the past five years. The top African importers of non-basmati (non-parboiled) rice in terms of the 2021–2022 average import quantity were Madagascar, Benin, Cameroon, Côte d’Ivoire, Kenya, Mozambique, Angola, Togo, and Guinea. Most major importers of non-basmati (non-parboiled) rice from Africa displayed a sizeable increase in imports from 2021 to 2022. In Asia, the major destinations of Indian non-basmati (non-parboiled) rice are Nepal, Vietnam, Malaysia, Bangladesh, Sri Lanka, and China. In India, the annual average volume of rice supply surplus was close to 11 million tons from 2005 to 2022 (Table 2). Indeed, the rice supply surplus enabled the India to export an average of 9.4 million tons of rice from 2005 to 2022 (Fig. 3 and Table 2). This volume represented 9% of total milled production and consumption on average over the same period.

**Table 1:** Non-basmati (non-parboiled) rice imports of the major trading partners of India, 2018–2022.

Region/country	2018	2019	2020	2021	2022	2021–2022	
						% Share	Rank
***Africa***							
Angola	2	4	29	114	373	4.4	10
Benin	51	35	208	350	533	8.0	3
Cameroon	1	1	66	244	334	5.2	5
Côte d'Ivoire	49	11	74	210	354	5.1	7
Guinea	135	47	130	219	164	3.5	12
Kenya	23	1	61	179	355	4.8	8
Madagascar	106	24	286	401	617	9.2	1
Mozambique	4	4	92	204	323	4.7	9
Togo	27	37	183	193	204	3.6	11
***Asia***							
Bangladesh	823	26	1	211	88	2.7	14
Malaysia	13	48	284	379	191	5.1	6
Nepal	465	384	570	599	374	8.8	2
People's Republic of China	1	1	2	48	136	1.7	16
Sri Lanka	34	3	0	33	181	1.9	15
Vietnam	0	0	26	333	298	5.7	4

**Table 2:** Milled rice production, consumption, and exports of India.

Year	Milled production (1000 mt)	Consumption (1000 mt)	Supply surplus (1000 mt)	Total exports (1000 mt)	Share of total exports to milled production	Share of total exports to total consumption
2005	83,127	80,858	2269	4569	5.50	5.65
2006	91,785	85,083	6702	4688	5.11	5.51
2007	93,345	86,695	6650	5740	6.15	6.62
2008	96,682	90,458	6224	4654	4.81	5.14
2009	99,172	91,082	8090	2090	2.11	2.29
2010	89,083	85,501	3582	2082	2.34	2.44
2011	95,970	90,196	5774	2774	2.89	3.08
2012	105,301	93,325	11,976	10,376	9.85	11.12
2013	105,241	93,972	11,269	10,869	10.33	11.57
2014	106,646	98,727	7919	10,619	9.96	10.76
2015	105,482	98,244	7238	12,238	11.60	12.46
2016	104,408	93,451	10,957	10,357	9.92	11.08
2017	109,698	95,838	13,860	11,710	10.67	12.22
2018	112,758	98,667	14,091	12,041	10.68	12.20
2019	116,484	99,164	17,320	10,420	8.95	10.51
2020	118,870	101,950	16,920	12,520	10.53	12.28
2021	124,368	101,052	23,316	20,216	16.25	20.01
2022	129,471	110,446	19,025	22,025	17.01	19.94
Average	104,883	94,151	10,732	9444	8.59	9.71

**Fig. 4:**
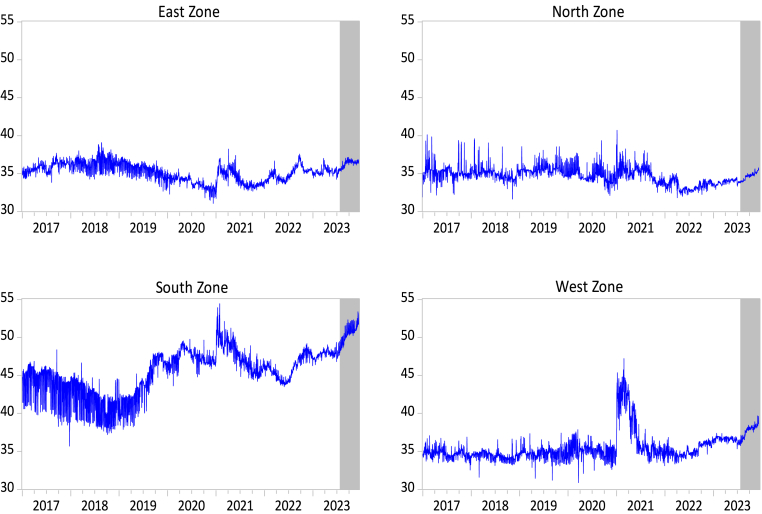
Daily rice retail prices in India by zone (real rupees per kg), January 1, 2017, to December 18, 2023. *Source of data:* Nominal retail prices are sourced from the Department of Consumer Affairs of India. CPI data are obtained from the IMF International Financial Statistics database.

### 2.2 Recent literature

Export restrictions, bans, taxes, or quotas have been implemented to ensure domestic food security, tax revenue, and price stabilization despite some repercussions. Although used for various reasons, the transmission effect and its distributive impact are a concern. In theory, an export ban increases the locally available supply, thus lowering consumer prices. In the international market, an export ban decreases the supply to the rest of the world, leading to higher global prices. The export ban widens the price gap between the implementing country and the rest of the world. As a small, open economy, the implementing country cannot influence the world market price unless the country is large enough. Then, the trading partner's price will increase. In both cases, the domestic price of implementing countries falls with the export ban (Porteous, 2017).

Several studies have examined the effect of cereal export restrictions on domestic prices. Aragie et al. (2018) used a static computable general equilibrium (CGE) to simulate the impact of a maize export ban in Malawi based on two scenarios.^3^ They showed that, in the short run, since the export ban diverted maize to local markets, the domestic supply increased by 6.8% and domestic maize prices declined by 15.5%. For the same commodity, Diao and Kennedy (2016) applied a dynamic CGE model for Tanzania and found that export bans lower maize consumer prices by 13–16%. In Malawi, Fuje and Pullabhotla (2020) used difference-in-difference regression to assess the short-term impact of export bans on the prices and welfare of Malawian households. The authors found that the net food buyers of maize had a positive but small surplus from low prices following the export ban, while the net sellers experienced a slightly larger welfare loss. Fellmann et al. (2014) investigated the temporary export restrictions on grain in Russia, Ukraine, and Kazakhstan using the AGLINK-COSIMO model based on a global recursive dynamic, partial equilibrium, and supply–demand framework. The authors showed that export restrictions would lower consumer prices below the prevailing market prices under normal weather conditions. Likewise, Woldie and Siddig (2009) simulated the overall effects of an export ban on wheat and other cereal crops in Ethiopia using the GTAP model. They showed that the export ban would reduce consumer prices by 13%. In contrast, Welton (2011) noted a different result in Russia, where the export control of grain did not bring down food prices inside the country but increased prices not only in the importing countries but also across the world. Moreover, Pham Van Ha et al. (2015) noted that the export ban hindered substantial rice sales in the international market at high world prices.

Aside from studies on prices, there were also empirical studies on the welfare effects of export control. The net welfare effect would depend, among others, on the relative share of net food buyers and sellers and the relative change in producers' and consumers' prices (Akter, 2022). In Vietnam, an analysis of rice export policy and export ban using the CGE model and a micro-simulation using household data showed significant distributional impacts on regional household welfare from different export policies and market conditions (Pham Van Ha et al., 2015). In a recent study, Aragie et al. (2018) showed that export bans benefit only the urban non-poor, while, in the long run, poor farmers’ income and consumption of maize decline. Similarly, Diao and Kennedy (2016), in analysing export control for maize using dynamic CGE, revealed a decrease in the wage rate for low-skilled labor and returns to land. However, the returns to non-agricultural capital and wage rates for skilled labor rise, further harming poor rural households and implying a greater poverty rate. Welton (2011) shared the same findings on higher poverty in Russia due to grain export control.

In terms of empirical investigation of the dynamic behavior of rice prices, Lee and Valera (2016) examined the price transmission and volatility spillovers in six major Asian rice markets (Bangladesh, China, India, the Philippines, Thailand, and Vietnam) from 2005 to 2013. Applying a panel GARCH model on monthly price data, they found that changes in the world rice price and the 2007–2008 price shocks increased both the domestic market's prices and their volatility. They also showed that interdependence across rice markets contributed to a strong spillover of a price shock from one country to another within Asia. Other studies have exploited the time dimensions of data in examining the relationship between world and domestic rice prices. John (2013), for example, investigated the price relations between export and domestic rice markets in Thailand using impulse response functions and found that, while the shocks originating in the domestic market are higher in magnitude in the export market in the short run, the shocks originating in the export market are more persistent in the domestic market. Furthermore, Minot (2014), who used monthly data on international grain prices in Africa over the period January 1980 to March 2011, showed that international grain prices had become more volatile in some years (2007–2010) but no evidence that food price volatility had increased in the region. From the perspective of the dynamic behavior of rice prices in the domestic market, Valera et al. (2022) estimated a panel vector auto-regression model using monthly prices for 17 regions in the Philippines from 2007 to 2019. They showed that rice price increases are a major source of inflation and that the response of inflation to a rice price shock varies according to poverty incidence by region.

As the above discussion suggests, the dynamic behavior of domestic rice prices has not been adequately examined from the perspective of imposing export restrictions in times of rising prices. To our knowledge, the dynamic behavior of domestic rice prices in India based on high-frequency data with a relatively long sample period remains to be seen. Thus, our study fills a potential gap in the existing literature by estimating the impact of India's export restrictions on the level and volatility of rice prices across different zones using the panel GARCH model.

## 3 Methodology and data

### 3.1 Methodology

We seek to estimate how India's export restrictions on non-basmati (non-parboiled) white rice affect the domestic retail prices of rice by building on the panel GARCH model proposed by Cermeño and Grier (2006).^4^ The panel GARCH model allows for both conditional and unconditional heteroskedasticity and conditional cross-sectional correlation of the error terms. The specifics of the dynamic panel model with fixed effects for a cross-section of *N* zones and *T* periods are presented in the equations below.

(1) $$\Delta \ln\left(P_{it}\right)=\mu_{i}+\sum_{j=1}^{12}{\beta_{k}P}_{it-j}+\beta_{13}{XR}_{t}^{\text{Ez}}+\beta_{14}{XR}_{t}^{\text{Nz}}+\beta_{15}{XR}_{t}^{\text{Sz}}+\beta_{16}{XR}_{t}^{\text{Wz}}+\varepsilon_{it}$$

(2) $$\sigma_{it}^{2}=\alpha_{i}+\delta {\;\sigma }_{i,t-1}^{2}+\gamma \varepsilon_{i,t-1}^{2}+\varphi_{1}{XR}_{t}^{\text{Ez}}+\varphi_{2}{XR}_{t}^{\text{Nz}}+\varphi_{3}{XR}_{t}^{\text{Sz}}+\varphi_{4}{XR}_{t}^{\text{Wz}}$$

(3) $$\sigma_{ijt}=\eta_{ij}+\lambda \sigma_{ij,t-1}+\rho \varepsilon_{i,t-1}\varepsilon_{j,t-1}\;\text{for}\;\text{each}\;\text{zone}\;\text{pair.}$$

The subscript *i* indexes regions or zones in India and *t* indexes time. The term $\mu_{i}$ captures possible time-invariant effects associated with retail prices of rice. The disturbance term $\varepsilon_{it}$ has a multivariate normal distribution with mean zero and time-varying variance-covariance matrix $\Omega_{t}$. The variance-covariance matrix $\Omega_{t}$ is a 4 × 4 symmetric matrix that is positive definite for all periods *t*. Equation (2) provides the four diagonal elements of $\Omega_{t}$ (i.e., the $\sigma_{it}^{2}$) while Equation (3) gives the six unique off-diagonal elements (i.e., the $\sigma_{ijt}$). The covariance specification follows Bollerslev (1990). Ez, Nz, Sz, and Wz are dummy variables representing the East Zone, North Zone, South Zone, and West Zone of India,^5^ respectively. In this way, we allow for zone-specific unconditional error variances and time-varying conditional cross-sectional dependence.

The variable ${XR}_{t}$ is a dummy variable taking the value of one during the implementation of export restrictions on non-basmati (non-parboiled) white rice from July 20 to December 18, 2023, and zero otherwise. The key coefficients testing for the effects on rice retail prices of non-basmati (non-parboiled) rice export restrictions are β and φ. If an export restriction effectively lowers the level and volatility of retail prices of rice, then both β and φ will be negative and significant.

### 3.2 Data

We performed model estimation using all-India average daily observations of domestic retail rice prices^6^ for four zones in India (*N =* 4) covering January 1, 2017, and December 18, 2023 (*T* = 2543). The sample consists of the East Zone, North Zone, South Zone, and West Zone. The data are obtained from India's Department of Consumer Affairs. The real domestic retail prices are calculated by dividing the daily nominal prices by the monthly consumer price index (CPI) data obtained from International Financial Statistics. The daily price observations provide us with a certain degree of richness using high-frequency rather than low-frequency data. For example, the main focus of the study is to examine whether or not retail prices of rice have declined following the export ban on non-basmati (non-parboiled) rice on July 20, 2023. Since India imposed export restrictions on non-basmati (non-parboiled) rice because of greater concern for ensuring domestic food security and low food prices, this impacts what happens after such a trade policy. Thus, analyzing a post-export restriction period is not difficult because of the adequate sample size from using daily data.

Fig. 4 plots the domestic retail prices of rice for each of the four zones in India. The figure illustrates that rice retail prices have increased rather than decreased since India imposed export restrictions on non-basmati (non-parboiled) rice on July 20, 2023. This is particularly true for the North Zone, South Zone, and West Zone, where rice retail prices continuously rose during the period July–December 2018. A similar pattern can be observed for the East Zone, although retail prices have stabilized since October 2023. However, the retail prices in all four zones remain higher than in the first six months of 2023. The pattern of a sudden shift in retail prices in 2021 can also be observed in Fig. 4, particularly in the East Zone, South Zone, and West Zone. The North Zone also experienced retail price surges in 2021, but the changes in domestic prices during that period were not as pronounced as in the other three zones. Overall, the series in Fig. 4 highlights a sudden shift among the levels of retail prices as well as varying price volatilities in different periods.

We also analyze the impact of India's non-basmati (non-parboiled) rice export restrictions on world rice prices and transmission to national rice economies. We use a weekly price series of Thai 5% broken white rice compiled from the USDA Foreign Agricultural Service GAIN Report. This enables us to provide more detailed accounts of the impact on Thai 5% prices from July 20, 2023, onwards. Regarding transmission to domestic rice prices, we analyze the percentage increase in retail or wholesale prices for a sample of Asian and African countries from August 2022 to July–October 2023, when India's export restrictions were put in place. The monthly data on retail or wholesale prices of rice are sourced from the FAO Global Information and Early Warning System on Food and Agriculture (GIEWS) Food Price Data and Analysis Tool. For Asian rice net exporters, we collected monthly retail price data for India (national average), Myanmar (rice price of Emata, medium) and Pakistan (average of basmati rice price in five markets). Wholesale rice price data were obtained for Cambodia (average price of rice mix in four markets), Thailand (price of Bangkok 25% broken rice), and Vietnam (average price of 25% broken rice in three markets). Likewise, we obtained a monthly retail price series for the net importing countries in Asia, such as Bangladesh (price of medium rice in Dhaka), Indonesia (national average price), Malaysia^7^ (national average price), Nepal (price of coarse rice in Kathmandu), and the Philippines (national average price of regular milled rice). As for Africa, we analyzed national monthly price data for Angola (average of long grain rice in eighteen markets), Benin (average of imported rice in three markets), Cameroon (average of retail price in five markets), Côte d’Ivoire (rice price in Abidjan market), and Togo (average of imported rice price in six markets), where data are available.

Asian and African countries are important samples to analyze because of white rice's increasing significance for the food security of those regions. In addition, Table 3 shows that most African sample countries have a high prevalence of moderate or severe food insecurity in the total population, reaching as high as 55.8% in Cameroon to 77.7% in Angola from 2019 to 2021. Moderate or severe food insecurity is also relatively high in Bangladesh, Nepal, and the Philippines. Therefore, assessing the implications of India's rice export restrictions on rice food security is an issue of paramount importance to the aforementioned countries in Asia and Africa.

**Table 3:** Prevalence of moderate or severe food insecurity in the total population.

	Prevalence of severe food insecurity in the total population (%)		Prevalence of moderate or severe food insecurity in the total population (%)		Share of rice calories to total calories (%)	
Country	2018–2020	2019–2021	2018–2020	2019–2021	2018–2020	2019–2021
*Africa*						
Angola	26.9	30.4	73.5	77.7	5.7	5.8
Benin	n/a	13.8	n/a	67.9	19.9	19.3
Cameroon	26.7	26.7	55.8	55.8	9.7	10.3
Côte d'Ivoire	n/a	9.4	n/a	42.8	24.4	23.9
Togo	n/a	18.8	n/a	62.5	6.6	8.3

*Asia*						
Bangladesh	10.5	10.7	31.9	31.7	67.6	67.0
Cambodia	13.4	15.1	44.8	50	54.8	53.7
Indonesia	0.7	0.7	6.2	6	40.6	38.9
Malaysia	7.5	6.3	18.7	15.4	23.6	23.9
Myanmar	1.9	3.7	22.2	25.5	47.1	51.8
Nepal	12	13.6	36.4	37.8	29.5	29.0
Philippines	4	4.8	42.7	43.8	41.7	42.2
Thailand	8.5	10.5	29.8	33.8	38.5	38.8
Vietnam	0.5	0.6	6.5	7.6	47.7	47.1

## 4 Results and discussion

### 4.1 Impacts on domestic rice prices

Before reporting the results, we want to know how likely a country is to implement export restrictions based on the exports-to-production ratio. Table 4 shows that Thailand and Pakistan have high exports-to-production ratios, indicating that their rice farmers depend highly on foreign markets. This explains why neither of those two countries implemented export restrictions during the 2008 rice crisis and have not done so this time. Indonesia's experience with palm oil is consistent with the above argument, as most of its palm oil is exported. Although Indonesia strengthened its export restrictions on palm oil earlier in 2023, it quickly lifted them because the damage to domestic farm prices was significant and farmers were concerned about their income and livelihood. Similarly, Myanmar, also announced restrictions on rice exports in 2023 but quickly remove them. On the other hand, India's and Vietnam's exports-to-production ratios are not as high as in Pakistan and Thailand. Thus, India imposed restrictions, which do not amount to a ban. Instead, the Indian government is taking temporary control of export decisions away from the private sector and deciding to engage in bilateral negotiations with rice-importing countries (Gozum, 2023). Vietnam imposed some export restrictions in the early phase of the COVID-19 pandemic, but the restrictions were relatively weak and were removed fairly quickly.

**Table 4:** Exports-to-production ratio for major Asian rice-exporting countries, 2005–2022.

Year	India	Vietnam	Thailand	Pakistan	Myanmar	Cambodia
2005	5.50	22.78	41.90	55.74	1.99	7.61
2006	5.11	20.66	40.53	66.05	0.45	11.53
2007	6.15	19.73	52.37	52.21	0.29	13.96
2008	4.81	19.07	50.56	53.60	4.57	8.86
2009	2.11	24.39	43.17	41.86	9.39	21.63
2010	2.34	26.94	44.65	58.11	6.01	18.71
2011	2.89	26.54	52.55	70.18	9.72	19.73
2012	9.85	28.42	33.94	56.10	11.83	19.41
2013	10.33	24.33	33.28	64.63	9.93	21.91
2014	9.96	22.46	53.61	58.11	14.12	20.16
2015	11.60	23.45	52.15	54.26	13.77	23.35
2016	9.92	18.45	62.60	61.75	10.69	21.29
2017	10.67	23.68	60.72	51.80	26.48	21.88
2018	10.68	23.83	54.49	53.84	20.83	23.41
2019	8.95	24.07	37.19	62.39	20.45	23.51
2020	10.53	22.76	32.37	53.01	18.18	23.52
2021	16.25	22.91	33.31	46.07	15.08	32.24
2022	17.01	26.45	38.65	51.67	18.83	29.46

Next, we begin our empirical work with data specification. Table 5 shows the unit-root test results for the log of daily retail price data series. A constant and trend were included in the unit-root tests. For the test results of the augmented Dickey-Fuller (1981) *t*-test and DF-GLS unit root test for individual time-series data reported in panel A of Table 5, optimal lag length is based on the Akaike Information Criterion (AIC). The results of the ADF-*t* test without and with one break^8^ show that retail prices expressed in log levels are non-stationary in most of the four zones. The only is East Zone where the unit root null is rejected at the 10% according to the ADF *t*-test with one break. With exception of the West Zone, the results of the conventional DF-GLS unit root test also show that the log of retail prices is non-stationary in the remaining three zones. Panel B of Table 5 also presents the corresponding panel unit-root tests for retail prices of rice. The null hypothesis is a unit root for the panel data for the Im-Pesaran-Shin (2003) and Levin-Lin-Chu (2002) tests. Both panel unit-root tests confirm that log of retail prices is non-stationary. Thus, the analysis in this study proceeds by using the data in first differenced or stationary form.

**Table 5:** Unit-root tests.

	ADF *t*-test		DF-GLS	Lee-Strazicich unit root test	
	No break	One break		LM test statistic	TB1, TB2
A) Time-series data					
East Zone	−2.30	−4.31*	−2.33	−0.098 (−6.592)***	August 22, 2018, January 11, 2021
North Zone	−3.01	−4.76	−1.67	−0.170 (−6.159) ***	November 11, 2018, April 3, 2022
South Zone	−1.84	−3.52	−2.00	−0.075 (−5.481)***	March 25, 2018, January 31, 2021
West Zone	−2.99	−3.43	−2.94**	−0.083 (−5.830)***	December 15, 2020, August 28, 2021
B) Panel data					
Im-Pesaran-Shin	−1.10				
Levin-Lin-Chu	1.36				

*Notes:* A constant and time trend are included in all regressions. The optimal lag for ADF *t*-test and DF-GLS is determined using the AIC. ** and * indicate rejection of the unit root null hypothesis at the 5% and 1% statistical levels, respectively. The ADF *t*-tests with one break are Perron's (1989; 1997) regressions, which allow for the presence of a one-time change in both the constant and the slope of the time trend. TB1 and TB2 are the dates of the structural breaks. Figures in parentheses are *t*-values. The 1%, 5% and 10% critical values for the minimum LM test with two breaks are −4.545, −3.842 and −3.504, respectively.

Next, results from applying the GARCH-type framework for understanding the volatility dynamics of rice prices are reported in Table 6. Results of both the Ljung-Box *Q* statistics for serial correlation and an *F*-test of ARCH effects in the residuals of regressions of first-differenced price series are based on 12 autoregressive lags, and relevant statistics are generated with a fourth-order autoregressive lag for the residuals. The results suggest that the autoregressive model of 12 lags is appropriate for all price series since the Ljung-Box *Q* statistics show scant evidence of serial correlation. Meanwhile, the ARCH test statistics provide strong evidence of conditional heteroskedasticity in the variances of the rice price data. Overall, the test statistics reported in Table 6 support the application of GARCH-type models for capturing the time-varying volatility dynamics in the Indian rice price series. This finding aligns with Lee and Valera (2016), who reported the leptokurtic nature of rice prices for a sample of Asian countries.

**Table 6:** Tests for serial correlation and conditional heteroskedasticity.

	Ljung-Box Q (4)	ARCH (4)
East Zone	1.15	26.12***
North Zone	1.71	35.48***
South Zone	3.75	52.05***
West Zone	0.45	101.25***

*Note:* ***p < 0.01.

Based on the patterns of the rice price data in the preceding section, it is difficult to generalize the conclusion on the impact on retail prices of rice of India's export restrictions on non-basmati (non-parboiled) white rice and volatility spillovers across regions or zones. This motivates the application of a panel regression framework outlined in Section 3. The results of the panel GARCH estimation are displayed in Table 7. In addition to the lagged values of the domestic prices, the panel GARCH estimates include the dummy variable controlling for India's export restrictions on non-basmati (non-parboiled) rice.

**Table 7:** Panel GARCH estimation results.

Conditional mean		Conditional variance		Conditional covariance	
Coefficient	Estimate	Coefficient	Estimate	Coefficient	Estimate
*Μ*	0.01 (0.39)	α_1_	−0.03 (24.69)***	η_21_	−0.01 (2.86)***
*P*_*i*_*, t*-1	−0.67 (38.56)***	α_2_	−0.08 (74.02)***	η_31_	0.03 (5.48)***
*P*_*i*_*, t*-2	−0.54 (25.92)***	α_3_	−0.05 (22.96)***	η_32_	0.001 (0.12)
*P*_*i*_*, t*-3	−0.47 (22.05)***	α_4_	−0.06 (29.70)***	η_41_	0.01 (2.08)**
*P*_*i*_*, t*-4	−0.39 (17.43)***	*XR*^*Ez*^	−0.007 (3.98)***	η_42_	−0.01 (2.50)**
*P*_*i*_*, t*-5	−0.36 (15.47)***	*XR*^*Nz*^	0.0002 (1.09)	η_43_	−0.01 (2.86)***
*P*_*i*_*, t*-6	−0.31 (18.38)***	*XR*^*Sz*^	0.002 (0.29)	Λ	0.95 (120.06)***
*P*_*i*_*, t*-7	−0.02 (4.64)***	*XR*^*Wz*^	0.004 (1.44)	Ρ	0.02 (8.16)***
*P*_*i*_*, t*-8	−0.07 (6.58)***	δ	0.97 (565.06)***		
*P*_*i*_*, t*-9	−0.09 (5.46)***	γ	0.03 (18.93)***		
*P*_*i*_*, t*-10	−0.08 (4.63)***				
*P*_*i*_*, t*-11	−0.08 (5.14)***				
*P*_*i*_*, t*-12	−0.05 (4.34)***				
*XR*^*Ez*^	0.06 (1.05)				
*XR*^*Nz*^	0.16 (4.61)***				
*XR*^*Sz*^	0.28 (3.33)***				
*XR*^*Wz*^	0.25 (3.85)***				

Log-likelihood	−18835.75				

*Notes:* Absolute values of t-statistics are in parentheses. ***p < 0.01 and **p < 0.05.

The first two columns of Table 7 contain the coefficient estimates for the conditional mean Equation (1). All coefficient estimates are statistically significant, except for the intercept term *μ* and the impact on retail prices in the East Zone of the dummy variable on India's non-basmati (non-parboiled) white rice export restrictions. The statistically insignificant estimate for intercept means the absence of a persistent upward or downward trend (i.e., no percentage change) in rice prices over the observation period. This finding is consistent with Lee and Valera (2016), who found a lack of a persistent upward or downward trend in domestic wholesale prices across a sample of six Asian rice-producing countries.

The most important results in Table 7 are the estimated values of the dummy variable on India's export restrictions on non-basmati (non-parboiled) rice, which is the key to judging the effectiveness of such an export policy in lowering domestic prices. A closer look provides insight into India's export restrictions on non-basmati (non-parboiled) rice, tend to increase rather than decrease the retail prices of rice in most of the four zones. In the conditional mean equation, the estimated coefficients for the *XR* variable are 0.16 for the North Zone, 0.28 for the South Zone, and 0.25 for the West Zone, all of which are significant at the 1% level. In other words, estimates suggest that this export restriction is associated with a 0.16%–0.28% increase in retail prices in the three zones. The estimated coefficient for *XR* in the East Zone's case is positive but insignificant. The above finding may seem counterintuitive, considering that India is an exporting country. There are several plausible explanations for this and not all may result the export restrictions. First, rice is storable, and India's export restrictions on non-basmati (non-parboiled) rice may have been perceived to be temporary (although such bans in the past have on occasion lasted several years); thus, traders can limit the actual effects of export restrictions by anticipating lifting of the ban through storage (see Gustafson, 1958; Deaton and Laroque, 1992). Thus, traders' higher volume in rice storage decreased the domestic rice supply in the domestic market, thus increasing domestic rice prices. Furthermore, it is not only traders who can engage in storage – farmers and consumers can do the same, with such speculative behavior leading to a surge in demand (Timmer, 2010). Second, expectations of adverse El Niño Southern Oscillation conditions continually worsened in the second half of 2023.^9^ In the past, these conditions have led to substantially reduced harvests for several Asian importers, so traders might have increasingly anticipated additional import demand in the second half of 2023.

The findings corroborate the evidence of increased consumer prices in Russia due to the imposition of a grain export ban, as reported by Welton (2011). On the other hand, the evidence of a significant positive impact on rice consumer prices of export restrictions does not concur with previous studies showing a reduction in maize consumer prices in Malawi (Fuje and Pullabhotla, 2020) and Tanzania (Diao and Kennedy, 2016), as well as in wheat prices in Russia (Fellmann et al., 2014) and Ethiopia (Woldie and Siddig, 2009). Most of these past studies relied on model-based analysis, but this technique fails to capture precisely the nature of possible changes in rice prices in the short term due to export restrictions. As mentioned earlier, we address this drawback by characterizing the impact of export restrictions on the dynamic behavior of daily rice prices in India. The third and fourth columns of Table 7 show the estimation results for the conditional variance Equation (2). The estimates for the *XR* coefficients are negative and statistically significant at the 1% level for the East Zone. The finding indicates that India's export restrictions lower the price variance in the East Zone. The coefficient estimates for *XR* are negative for the North Zone and West Zone and positive for the South Zone, but these are insignificant. The results further show that the parameter estimate for δ is at 0.96, reflecting a high persistence in price volatility. The estimate for the parameter *γ* of 0.03 indicates the positive influence of a price shock on domestic price volatility. The coefficient estimates for the conditional covariance in Equation (3) are shown in the fifth and sixth columns of Table 7. Most of the estimates for *η*_ij_ (*i*≠*j*) are statistically significant and negative, which means that the variances of the four domestic rice prices vary in the opposite direction over time. We also find a high persistence among the conditional covariances, as indicated by the λ with an estimated coefficient of 0.98. The estimate for ρ is also statistically significant, supporting the impact of a price shock on the conditional covariances.

### 4.2 Impacts on world rice prices and transmission to national rice economies

We have so far uncovered two key findings. First, India's export restrictions on non-basmati (non-parboiled) rice coincided with rising domestic retail prices of rice in three zones: North, South, and West. Second, for the East Zone, there is evidence of a reduction in the volatility of retail prices following India's export restrictions. In further exploring the impact on third countries, we now assess the extent of vulnerability of exporting and importing countries from global rice price fluctuations created by India's export restrictions. This analysis builds upon earlier studies such as Dawe, 2008, Dawe, 2009, and Dawe and Kimura (2023) that have examined international price transmission to domestic rice markets. Such an analysis is worthwhile because, as Dawe and Kimura (2023) emphasized, the world rice market is very important for food security since it provides a supply of rice that can offset fluctuations in domestic production and thereby help to stabilize domestic prices. Dawe and Kimura (2023) explained that global price fluctuations can be a source of price shocks if a country is open to international rice trade. That is why this possibility has been a traditional concern of Asian policymakers. The world rice market is an important potential stabilizing force, but it indirectly affects food security as poor consumers purchase their rice on domestic markets at domestic prices (Dawe and Kimura, 2023). Hence, food security concerns arise only if transmission to domestic markets occurs.

**Fig. 5:**
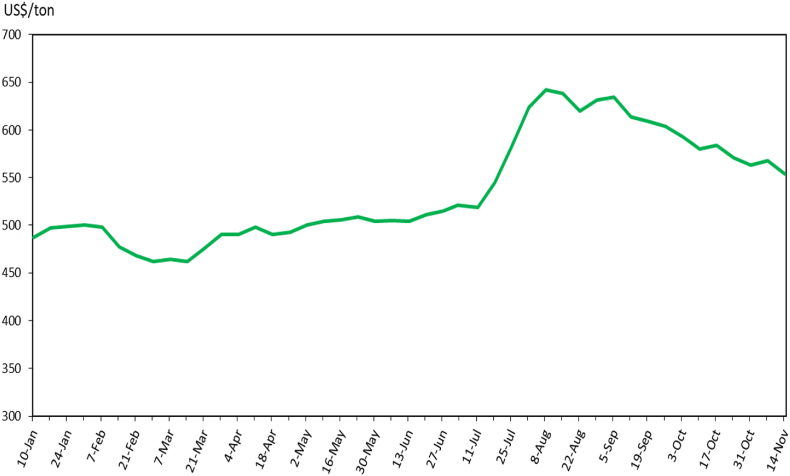
Impacts on weekly prices of Thai 5% white rice, January–November 2023. *Source:* USDA GAIN Attaché Reports. *Note:* The shaded area represents the period of non-basmati (non-parboiled) rice export restriction.

Before analyzing the international price transmission to national economies, examining the impact on world rice prices first is important. Fig. 5 shows the weekly prices of Thai 5% white rice from January 10 to November 14, 2023. The price of Thai 5% white rice increased substantially in the three weeks after India's export restrictions on non-basmati (non-parboiled) rice. In particular, the Thai 5% white rice price rose by nearly USD 60 per ton from July 20 to August 8, 2023. This constitutes a substantial increase in a short period of time, suggesting there was a market reaction to the export restrictions but not a panic similar to that in 2008, when world prices tripled in the span of just a few months. Because of political pressure from other countries and food insecurity, the markets sensed that the export restriction on non-basmati (non-parboiled) white rice was temporary because the Indian government started bilateral trade deals (or direct deals) with importing countries, with pre-approved government agencies or contractors. Over the past several months, the Indian government has directly sold rice to Indonesia, Senegal, Gambia, Mali, and Ethiopia. Starting in mid-August, Thai 5% white rice prices decreased, reaching USD 554 per ton on November 14, 2023. However, Fig. 5 shows that the weekly price of Thai 5% white rice is significantly higher in the past two months compared to the January to July period in 2023.

**Fig. 6:**
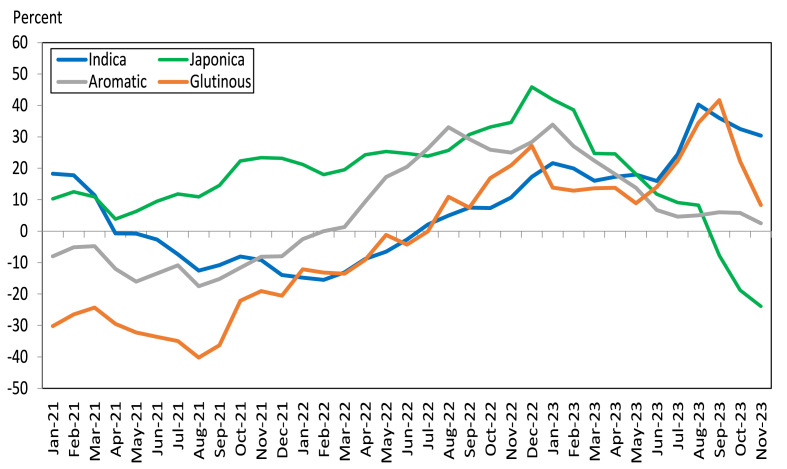
Year-on-year percentage increase in FAO international rice price index. *Source of raw data:* FAO Food Price Monitoring Tool.

In furthering our understanding of international transmission to domestic rice prices, it is important to examine which submarkets of the world rice market have a greater concern for food security implications in times of global rice price fluctuations. These submarkets refer to Indica rice (long and slender grain grown in the tropics and subtropics), Japonica rice (short and round grain grown in the subtropics and temperate zones), and aromatic rice such as jasmine from Thailand and basmati from India and Pakistan (Valera et al., 2023). Plots of the monthly price indices are presented in Fig. 6 for the Indica, Japonica, aromatic, and glutinous rice markets. As can be seen, the plots of price indices of these different submarkets often move in different directions. Note that price increases in the Japonica and aromatic submarkets have less regard for food security implications than price increases in the Indica submarket. This is because aromatic rice is relatively expensive and primarily consumed by higher-income consumers. In contrast, Japonica rice is eaten mainly in Japan, the Republic of Korea, and northern China, where much of this region is not poor (Dawe and Kimura, 2023). The same is true when comparing the food security implications of price increases between the Indica and glutinous rice submarkets. Glutinous rice is mainly consumed during holidays. However, glutinous rice is consumed as a daily staple throughout the year in Lao PDR and parts of Thailand.

As depicted in Fig. 6, the FAO Indica price index increased substantially since August 2022 by nearly 5% and continuously rose to as much as 22% until January 2023. Although the FAO Indica price index declined from February to June 2023, it increased sharply when India restricted non-basmati (non-parboiled) rice exports in the third week of July 2023. The FAO Indica price index increased to as much as 40% in August 2023. The discussion above suggests that international price transmission to domestic rice prices in Asia and Africa can be accomplished by calculating the cumulative percentage increase in prices from August 2022 until July–October 2023, when India imposed export restrictions and where price data are available for the countries under review.^10^ Table 8 reports the cumulative percentage increase in the retail prices of rice in selected Asian and African countries. The second to sixth columns of Table 8 report the percentage change in the nominal world price in local currency (LC) terms, as mediated by the exchange rate for each country. Exchange rate movements were small in most countries from August 2022 until October 2023. The most noticeable exceptions are Pakistan and Angola, where the exchange rate depreciated from 221 rupees per dollar and 230 kwanza per dollar in August 2022 to 281 rupees per dollar and 826 kwanza per dollar in October 2023, respectively. This means that such exchange rate depreciation has considerably magnified the change in the world price for the people and economy of Pakistan and Angola.

**Table 8:** Cumulative percentage increase in rice prices from August 2022 to June–October 2023.

	World price LC in 2023					Domestic price LC in 2023				
	Jun	Jul	Aug	Sept	Oct	Jun	Jul	Aug	Sept	Oct
**A. Asian exporters**										
Cambodia	19.86	27.84	48.92	45.00	36.55	−0.34	2.36	7.74	7.74	19.60
India	23.27	31.22	53.54	50.37	41.90	6.17	8.82	10.87	12.66	13.79
East Zone						−0.86	−0.03	−0.10	2.97	3.05
North Zone						3.52	2.66	2.88	5.21	5.52
Northeast Zone						18.77	19.74	19.85	24.08	23.52
South Zone						3.78	4.77	4.52	9.36	10.34
West Zone						4.51	3.59	3.12	8.44	9.08
Myanmar	19.26	26.93	47.54	44.04	35.62	62.98	78.64	90.57	93.01	81.01
Pakistan	54.84	61.90	96.58	94.13	72.29	76.10	81.68	85.11	88.06	87.39
Thailand	16.04	22.43	44.13	43.88	38.05	13.79	20.96	45.43	45.58	40.76
Vietnam	22.20	30.82	52.93	49.56	39.60	27.59	33.23	65.54	60.20	77.70
**B. Asian importers**										
Bangladesh	33.05	45.57	70.17	66.91	57.90	−3.07	−2.90	−4.55	−0.88	2.79
Indonesia	19.91	28.65	51.46	48.93	43.76	12.20	12.18	13.78	20.17	22.23
Malaysia	23.73	30.85	52.31	50.93	44.14	35.08	35.08	28.59	50.67	35.08
Nepal	22.85	31.38	51.99	50.04	41.73	16.00	26.00	28.00	36.00	40.00
Philippines	19.56	25.14	48.63	46.72	38.15	3.68	6.66	12.14	22.15	16.70
**C. Africa**										
Angola	97.95	143.63	183.28	176.68	160.80	4.99	6.57	9.58	12.23	15.64
Benin	11.43	16.35	36.99	36.56	30.05	1.81	1.57	1.99	1.87	1.81
Cameroon	11.43	16.35	36.99	36.56	30.05	3.30	3.30	4.18	5.03	6.10
Côte d’Ivoire	11.43	16.35	36.99	36.56	30.05	3.76	5.45	8.27	12.03	9.77
Togo	11.43	16.35	36.99	36.56	30.05	4.34	5.21	5.73	5.73	5.56

*Notes:* World price refers to Thai 5% broken for all countries. All price changes are in nominal terms.

The seventh to ninth columns of data in Table 8 present the percentage change in domestic rice prices. These changes are generally less than the percentage change in the world price in LC terms. According to Dawe and Kimura (2023), the smaller percentage change in domestic prices could be driven by the policies implemented by many countries to reduce the transmission of world prices to domestic markets, making the food security implications less strong. Of the world's six largest exporters, the changes in the world and domestic prices in Pakistan and Thailand are somewhat closer for August–October 2023. One potential explanation for this result is that these two countries do not have policies that generate large bands between world and domestic prices. In the case of India, the government imposed a 20% tax on exports of non-basmati (non-parboiled) rice in September 2022 and restricted non-basmati (non-parboiled) rice in July 2023. These policies have led to lower domestic price increases relative to world prices, as seen in Table 8. Indian domestic retail rice prices increased nonetheless from July to October 2023 (from 8% to 13%) based on the price data reported by FAO. However, at the zone level, retail prices declined slightly (0.03%–0.10%) in the East Zone in July and August 2023, according to the data obtained from India's Department of Consumer Affairs. Retail prices in the other four zones increased during the July–October 2023 period. The increase in retail rice prices is more pronounced in the Northeast Zone (19%–24%) and South Zone (3%–9%). The cumulative percentage price increases are consistent with the panel GARCH results mentioned earlier.

Overall, the data in Table 8 provide complementary information on how India's recent export restrictions likely contributed to higher retail rice prices worldwide in many domestic rice markets. For instance, there have been substantial domestic price increases in some countries, particularly exporting countries such as Myanmar (from 78% to 93%), Pakistan (81%–88%), Vietnam (33%–77%), and Thailand (20%–45%). In other words, exporters are the most vulnerable to global rice price fluctuations due to India's export restrictions.^11^ Exporters can reduce the risk, but mainly because they can restrict exports. If exporting countries do not restrict exports, they are very vulnerable. Poor countries (e.g., Cambodia and Myanmar) are particularly more vulnerable than countries with relatively higher GDP per capita (e.g., Thailand).

Finally, among rice-importing countries, substantial rice price increases of more than 26% are more pronounced in the cases of Malaysia and Nepal during the period July–October 2023. From August to October 2023, domestic rice prices in Indonesia and the Philippines increased substantially by more than 12%. The only exception to such a pattern of increasing domestic rice prices is Bangladesh, where retail prices declined from July to September 2023. Generally speaking, in countries where the private sector is deciding on rice import quantities (e.g., the Philippines, Bangladesh), international price transmission to domestic prices should occur, although exchange rate movements also have an impact. Implementing price caps for regular and well-milled rice in the Philippines from September to October 2023 has made the country more vulnerable because private traders become reluctant to import. Rice-importing countries where the government makes import quantity decisions (e.g., Indonesia) are also vulnerable to global rice price fluctuations. In broad terms, the price increases should be less than for importers in countries where the private sector makes import quantity decisions. Still, typically, that is because domestic rice prices are quite high even in normal times. Because they start so high, they increase less in the case of a price increase in world rice markets.

## 5 Conclusions

We employ a novel database of the domestic daily retail prices of rice for four zones in India over a seven-year study period to provide new insights into the dynamics of rice prices in the short run. Focusing on the impact of India's export restrictions on non-basmati (non-parboiled) rice, we conclude that such a trade policy was not sufficient enough in leading to lower domestic rice prices, which was a key goal of the policy. More specifically, by examining the conditional mean of daily retail prices of rice in the panel GARCH setting, results from this study suggest that India's export restrictions coincided with increased domestic prices in most of the four zones. We also uncovered a reduction in rice price variance in the East Zone, suggesting that India's export restrictions could have had, to a limited extent, the desired correlation with rice price volatility. Additionally, we showed that exporting and importing countries are vulnerable to global rice price fluctuations due to the turmoil in the world rice market created by India's export restrictions. However, the degree and kinds of vulnerability differ across exporters and importers.

Several policy implications emerged from our findings. First, one would ideally like to see a free flow of rice across countries or regions as that will provide a necessary cushion for the market against supply or demand shock. Rice-importing countries should keep diplomatic channels open with rice-exporting countries, particularly Vietnam, Cambodia, and Myanmar. For example, governments of importing countries should emphasize the advantages to Vietnam. The main advantage of Vietnam not employing an export ban is that it benefits their farmers, just like the higher prices will benefit rice farmers in rice-growing and -importing countries. For Vietnam, not employing an export ban is an opportunity for its farmers because they often get very low prices. If Vietnam employs an export ban, rice farmers will be hurt. Indonesia has had a similar experience since it had to repeal its export ban on palm oil because the ban was very restrictive and farmers were upset. Vietnam had its own experience in 2020 when the farmers were unhappy about temporary export restrictions because of COVID-19 in April 2020. From a policy perspective, finding here underscores the importance emphasizing trade policies that do not restrict rice exports.

The other policy implication, which is separate from gaining market share, is that it helps the rice importing countries if Vietnam and other exporting countries keep their market open because it makes the world rice market larger. The imposition of export ban among rice-exporting countries would lower their market share while rice-importing countries would be forced to be more self-sufficient. This in turn would make the world market smaller. Keeping rice trade open can create a bigger market for all rice-exporting countries, creating an opportunity for them to grow their market share, benefit their farmers and increase food security.

Another policy option to manage rice price instability is to consider income support to the poor via existing social safety nets if domestic rice prices rise substantially. The poorest consumers and most vulnerable households would be protected with adequate safety nets when a rice price increase threatens food security. A suitable approach in this direction would be to expand existing cash transfer programs. Policymakers, however, would have to ensure that safety nets are well-targeted to the poor and are endowed with enough fiscal resources. In terms of domestic production, the first thing to be done is to monitor domestic rice prices. Aside from monitoring domestic prices, it is also important to monitor the domestic weather forecast in terms of actually helping farmers. Another measure is to organize the early distribution of seeds for the upcoming dry season. This means that rice planting should be done early with some residual soil moisture that allows the rice plants to use it. In this case, one option is for farmers to use shorter-duration rice varieties and rice varieties that are more drought-tolerant. Finally, considering that the timing of India's rice export restrictions coincides with the concern about climate risks associated with El Niño, it is important that the government get people to share water better between upstream and downstream users. For example, the government might consider supporting some upstream farmers: not to give up all upstream water but to share it with some of the people downstream in the irrigation system. This is important because, if farmers can share irrigation water better, they will have more area planted.

## CRediT authorship contribution statement

**Harold Glenn A. Valera:** Writing – review & editing, Writing – original draft, Visualization, Software, Resources, Methodology, Investigation, Formal analysis, Conceptualization. **Ashok K. Mishra:** Writing – review & editing, Writing – original draft, Investigation, Formal analysis, Conceptualization. **Valerien O. Pede:** Writing – review & editing, Resources, Methodology, Funding acquisition, Formal analysis, Conceptualization. **Takashi Yamano:** Writing – review & editing, Investigation, Funding acquisition, Conceptualization. **David Dawe:** Writing – review & editing, Methodology, Formal analysis.

## Declaration of competing interest

The authors declare that they have no known competing financial interests or personal relationships that could have appeared to influence the work reported in this paper.

## Data Availability

Data will be made available on request.

## Footnotes

1 Milled or white rice is defined as husked rice wherein a portion of the bran and germ has been disconnected by milling (de Oliveira et al., 2020).

2 Parboiled rice can be either brown rice or white rice that is water-immersed and heat-treated to obtain a completely gelatinized starch succeeded by a drying process (de Oliveira et al., 2020).

3 Scenario one is the short-run scenario, under which local production is restricited to equal the sum of the base-level exports and domestic supply, i.e., there are no supply responses from producers. The second scenario is the longer-run scenario, which assumes that maize producers have the option to reallocate resources away from maize production to more profitable crops, subject to overall land constraints.

4 Recent studies that employed the panel-GARCH methodology include, among others, Lee (2010), Cermeño et al. (2010), Goulas and Zervoyianni (2013), Drakos and Konstantinou (2013), Escobari and Lee (2014), Cermeño and Sanin (2015), Lee and Valera (2016), Rezitis and Tremma (2023), Valera et al. (2016), and Valera et al. (2018).

5 The Northeast Zone was not included in this study because of several unreported daily rice price observations.

6 The data collected by India's Department of Consumer Affairs are average prices among 550 market centers split into five zones: North, West, East, Northeast, and South.

7 Price data for Malaysia is unavailable in FAO GIEWS thus data was sourced from online news articles.

8 In the case of the presence of multiple breaks, Table 5 also reports the data-driven breaks identified by the Lee and Strazicich (2003) unit root test.

9 In mid-July 2023, the National Oceanic and Atmospheric Administration (NOAA) forecasted a 20% chance of an historically strong El Niño (seasonally averaged El Niño 3.4 ≥ 2.0 °C). By mid-December, NOAA forecasted a 54% chance of such an event.

10 Of course, there are many factors that affect domestic price formation other than price transmission from world markets (e.g., input costs, prices of substitute outputs, weather conditions), and these idiosyncratic factors may account for some part of the different price changes in different countries. The analysis reported here is unable to control for this myriad of factors, but the widespread nature of the price increases does suggest some role for India's export restrictions. This is all the more so because world fertilizer, wheat and maize prices were all declining in 2023.

11 Exporting countries are, strictly speaking, not vulnerable, as they benefit from higher prices. It is the poor citizens who are net rice consumers in exporting countries who are vulnerable.

## References

[bib1] Abbott P.C. (2011). Export restrictions as stabilization responses to the food crisis. Am. J. Agric. Econ..

[bib2] Akter S. (2022). The effects of food export restrictions on the domestic economy of exporting countries: A review. Global Food Secur..

[bib3] Anderson K., Nelgen S. (2012). Agricultural trade distortions during the global financial crisis. Oxf. Rev. Econ. Pol..

[bib4] Aragie E., Pauw K., Pernechele V. (2018). Achieving food security and industrial development in Malawi: Are export restrictions the solution?. World Dev..

[bib5] Bollerslev T. (1990). Modelling the coherence in short-run nominal exchange rates: multivariate generalized ARCH model. Rev. Econ. Stat..

[bib6] Cermeño R., Grier K.B., Baltagi B.H. (2006).

[bib7] Cermeño R., Sanin M.E. (2015). Are flexible exchange rate regimes more volatile? Panel GARCH evidence for the G7 and Latin America. Rev. Dev. Econ..

[bib8] Cermeño R., Grier K., Grier R. (2010). Elections, exchange rates and reform in Latin America. J. Dev. Econ..

[bib9] Dawe D. (2008). Lost transmission. Rice Today.

[bib10] Dawe D. (2009). Cereal price transmission in several large Asian countries during the global food crisis. Asian Journal of Agriculture and Development.

[bib11] Dawe D., Kimura S. (2023). Development Asia.

[bib12] de Oliveira M., Ferreira C.D., Lang G.H., Rombaldi C.V., Costa de Oliveira A., Pegoraro C., Ebeling Viana V. (2020). The Future of Rice Demand: Quality beyond Productivity.

[bib13] Deaton A., Laroque G. (1992). On the behaviour of commodity prices. Rev. Econ. Stud..

[bib14] Diao X., Kennedy A. (2016). Economy-wide impact of maize export bans on agricultural growth and household welfare in Tanzania: A dynamic computable general equilibrium model analysis. Dev. Pol. Rev..

[bib15] Drakos K., Konstantinou P.T. (2013). Investment decisions in manufacturing: Assessing the effects of real oil prices and their uncertainty. J. Appl. Econom..

[bib16] Escobari D., Lee J. (2014). Demand uncertainty and capacity utilization in airlines. Empir. Econ..

[bib17] FAO, IFAD, UNICEF, WFP, WHO. (2021).

[bib18] FAO, IFAD, UNICEF, WFP, WHO. (2022).

[bib19] Fellmann T., Hélaine S., Nekhay O. (2014). Harvest failures, temporary export restrictions and global food security: the example of limited grain exports from Russia, Ukraine and Kazakhstan. Food Secur..

[bib20] Fuje H., Pullabhotla H.K. (2020). Impact of grain trade policies on prices and welfare: evidence from Malawi. The World Bank Poverty and Equity Global Practice Unit Policy Research Working.

[bib21] Goulas E., Zervoyianni A. (2013). Economic growth and crime: does uncertainty matter?. Appl. Econ. Lett..

[bib22] Gozum I. (2023). Philippines gets highest rice export allocation from India. https://www.rappler.com/nation/philippines-gets-highest-rice-export-allocation-india/.

[bib23] Gustafson R.L. (1958).

[bib24] Headey D.D. (2011). Rethinking the global food crisis: the role of trade shocks. Food Pol..

[bib25] John A. (2013). Price relations between export and domestic rice markets in Thailand. Food Pol..

[bib26] Lee J. (2010). The link between output growth and volatility: evidence from a GARCH model with panel data. Econ. Lett..

[bib27] Lee J., Strazicich M. (2003). Minimum LM unit root test with two structural breaks. Rev. Econ. Stat..

[bib28] Lee J., Valera H.G.A. (2016). Price transmission and volatility spillovers in Asian rice markets: evidence from MGARCH and panel GARCH models. Int. Trade J..

[bib29] Liefert W.M., Westcott P., Wainio J. (2011). Alternative policies to agricultural export bans that are less market-distorting. Am. J. Agric. Econ..

[bib30] Martin W., Anderson K. (2011). Export restrictions and price insulation during commodity price booms. Am. J. Agric. Econ..

[bib31] Minot N. (2014). Food price volatility in sub-Saharan Africa: has it really increased?. Food Pol..

[bib32] Mitra S., Josling T. (2009). Agricultural export restrictions: welfare implications and trade disciplines. International Food Agriculture. Trade Policy Council.

[bib33] Mohanty S. (2023). Indian rice export ban could bring about major changes in global rice market. Food Secur..

[bib34] Pham Van Ha P.V., Nguyen H.T.M., Kompas T., Tuong Nhu Che T.N., Trinh B. (2015). Rice production, trade and the poor: regional effects of rice export policy on households in Vietnam. J. Agric. Econ..

[bib35] Porteous O. (2017). Empirical effects of short-term export bans: the case of African maize. Food Pol..

[bib36] Rezitis A.N., Tremma O.A. (2023). The linkage between international dairy commodity prices and volatility: a panel-GARCH analysis. J. Agribus. Dev. Emerg. Econ..

[bib37] Sankaranarayanan S., Zhang Y., Carney J., Nigussie Y., Esayas B., Simane B., Zaitchik B., Siddiqui S. (2020). What are the domestic and regional impacts from Ethiopia's policy on the export ban of teff?. Front. Sustain. Food Syst..

[bib38] Sharma H. (2023). The Country.

[bib39] Timmer C.P., Dawe D. (2010). The Rice Crisis: Markets, Policies and Food Security.

[bib40] Valera H.G., Holmes M.J., Hassan G.M. (2016). Stock market uncertainty and interest rate behaviour: a panel GARCH approach. Appl. Econ. Lett..

[bib41] Valera H.G., Holmes M.J., Hassan G.M. (2018). Is inflation targeting credible in Asia? A panel GARCH approach. Empir. Econ..

[bib42] Valera H.G., Balié J., Magrini E. (2022). Is rice price a major source of inflation in the Philippines? A panel data analysis. Appl. Econ. Lett..

[bib43] Valera H.G.A., Holmes M., Pede V.O., Balié J. (2023). How convergent are rice export prices in the international market?. Agric. Econ..

[bib44] Welton G. (2011). The imp act of Russia's 2010 grain export ban. Oxfam Research Reports. Oxfam Policy and Practice: Agriculture. Food and Land.

[bib45] Woldie G.A., Siddig K. (2009). The impact of banning export of cereals in response to soaring food prices: evidence from Ethiopia using the new GTAP African database. http://mpra.ub.uni-muenchen.de/18241/1/MPRA_paper_18241.pdf.

